# CD133/prominin1 is prognostic for GBM patient’s survival, but inversely correlated with cysteine cathepsins’ expression in glioblastoma derived spheroids

**DOI:** 10.2478/v10019-011-0015-6

**Published:** 2011-06-03

**Authors:** Seyed Y. Ardebili, Irena Zajc, Boris Gole, Benito Campos, Christel Herold-Mende, Sara Drmota, Tamara T. Lah

**Affiliations:** 1 Department of Neurosurgery, University Medical Centre, Ljubljana, Slovenia; 2 Department of Genetic Toxicology and Cancer Biology, National Institute of Biology, Ljubljana, Slovenia; 3 Division of Neurosurgical Research, Department of Neurosurgery, University of Heidelberg, Heidelberg, Germany; 4 Department of Chemistry and Biochemistry, Faculty of Chemistry and Chemical Technology, University of Ljubljana, Ljubljana, Slovenia

**Keywords:** CD133/prominin1, cysteine cathepsins, glioblastoma, glioma stem cells, invasion, neural stem cells

## Abstract

**Introduction:**

CD133 is a marker for a population of glioblastoma (GBM) and normal neural stem cells (NNSC). We aimed to reveal whether the migratory potential and differentiation of these stem cells is associated with CD133 expression and with cathepsin proteases (Cats).

**Materials and methods.:**

The invasiveness of normal NNSC, GBM/CD133+ cell lines and GBM spheroids was evaluated in 3D collagen, as well as of U87-MG and normal astrocytes (NHA) grown in monolayers in 2D Matrigel. Expression of Cats B, L and S was measured at mRNA and activity levels and their relation to invasiveness, to CD133 mRNA in 26 gliomas, and to the survival of these patients.

**Results:**

The average yield of CD133+ cells from GBM samples was 9.6 %. Survival of patients with higher CD133 mRNA expression was significantly shorter (p< 0.005). Invasion, associated with proteolytic degradation of matrix, was higher in normal stem cells and GBM spheroids and cells than in isolated GBM CD133+ cells. In glioma samples, there was no correlation between CD133 mRNA expression and Cat mRNAs, but there was an inverse correlation with Cat activities.

**Conclusions:**

The study confirms CD133 as a prognostic marker for the survival of GBM patients. We demonstrated that NNSC have higher invasion potential and invade the collagen matrix in a mode different from that of GBM, initiating stem cell spheres. This result could have implications for the design of new therapeutics, including protease inhibitors that specifically target invasive tumour stem cells. Increased activity of cathepsins in CD133– cells suggests their role in the invasive behaviour of GBM.

## Introduction

Gliomas are the most abundant brain tumours, progressing from benign astrocytomas *via* anaplastic astrocytomas to the most malignant form, glioblastoma multiformae (GBM). The poor prognosis and short life expectancy for GBM patients is partly related to the high invasiveness of the tumour cells. In contrast to carcinoma, GBM cells infiltrate the normal brain parenchyma as single cells, making this tumour extremely difficult to target by conventional therapy.^1^–^3^ GBM is highly heterogenous, consisting of various types of cells. According to the hierarchical model of tumourigenesis, only a small fraction of tumour cells, the cancer stem cells (CSC), are capable of initiating tumour growth, and renewing the tumour in the same or other organ after incomplete surgical removal.^4^–^6^ When injected orthotopically, these cells were phenotypically characterised as capable of self renewal, asymmetric division and tumour formation in animal models of the same growth characteristics. These cells are also highly resistant to chemo- or radio-therapy^6^, ^7^ and presumably they and/or their immediate progenitors have high invasive potential to seed at a distance from the tumour.^8^

In a selective GBM stem cell population, plasma membrane associated protein CD 133/prominin-1 is considered as a cell surface marker of stemness and has been widely used for identifying putative stem cells from a variety of untransformed and cancerous tissues. However, CD133 is also expressed in differentiated epithelial cells in various organs, as well as in hematopoietic cells.^9^ From its first use for identification of cancer stem cells in brain tumours^10^, CD133 is still the most commonly used brain cancer stem cell marker, despite the many contradictions regarding the methods used to detect the expression of a surface marker in brain tumours. Some studies have shown that not all high grade gliomas express CD133^11^ and also, that CD133 negative cell populations from GBM may have tumour initiating potential^12^, giving rise to CD133+ tumours.^13^–^15^ The role of this marker in further steps of tumour progression is not known.

Cancer stem cells are, presumably, not only associated with high resistance to therapy but also with higher invasion and metastatic potential, as proposed by Brabletz *et al.*^8^ Proteolytic enzymes, including lysosomal cathepsins, participate in many normal and pathological processes and have been associated with cancer progression, mostly with invasion.^16^–^19^ Cysteine cathepsins B, L and S (CatB, CatL and CatS) and the aspartic cathepsin D are over-expressed in many tumour tissues and cells, and have been reported to be mediators of glioma invasion.^19^–^21^ Cysteine cathepsins comprise the largest family of lysosomal enzymes, with 11 proteases structurally grouped in CatB-like and CatL-like enzymes (http://www.merops.ec.uk).^22^ We have demonstrated the prognostic impact of CatB, but not of CatL, on survival of glioma patients.^23^,^24^ *In vitro*, we have recently confirmed the inhibition of the invasion of permanent GBM cell lines, as well as primary GBM spheroids by synthetic CatB inhibitors, emphasizing the role of CatB activity that was induced posttranslationally in the invasive GBM subpopulation.^25^

Although the homologous enzyme, CatL, is also correlated with glioma progression^26^–^28^, it appeared to be more relevant to proliferation and apoptosis than to the invasion process.^29^ Flannery *et al*.^30^ demonstrated that expression of CatS was an independent predictor of survival in GBM tumours, presumably also being related to invasion. However, the proteolytic efficacy of cysteine cathepsins is regulated at all levels of their expression, ultimately by their endogenous inhibitors, the cystatins (http://www.merops.ec.uk).^22^ Cystatin superfamily comprises two different families, cystatin family (with extracellular cystatins) and stefin family (with intracellular stefins), all these playing a role in cancer progression.^17^,^31^ A specific role for lysosomal cathepsins in stem cells biology has not been reported.

The first aim of the present study was to demonstrate CD133 mRNA expression in cancerous and normal neurospheres and GBM primary spheroids, and to assess whether there is any prognostic value of this marker for GBM patients treated with standard therapeutic protocols. Secondly, we aimed to establish whether there is any correlation between CD133 and the lysosomal cysteine cathepsins, CatB, CatL and CatS and their inhibitors stefin B and cystatin C, at various levels of expression in these tumours. Finally, we were interested in correlation between proteolysis and the invasion of a variety of CD133 expressing normal and cancerous cells under *in vitro* conditions.

## Materials and methods

### Glioblastoma patients

The patients were operated at the Department of Neurosurgery, University Clinical Centre of Ljubljana, Slovenia. Tumour samples were collected from 26 patients (16 male, 10 female, median age 60 years, Table 1). 24 patients were diagnosed with WHO grade IV glioblastoma and the remaining two with WHO grade III anaplastic astrocytoma by standard histopathology protocols at the Institute of Pathology, Faculty of Medicine in Ljubljana. These patients were all treated by standard protocols as shown in Table 1. The study was approved by the National Medical Ethics Committee of the Republic of Slovenia (Approval no. 109, 204-6/10/07).

### Tumour samples and primary tumour culture preparation

Immediately after removal from the patients, the tumour samples were placed in sterile ice-cold “stem cell buffer” (124 mM NaCl, 5.0 mM KCl, 1.3 mM MgCl_2_, 2.0 mM CaCl_2_, 26 mM NaHCO_3_, 10 mM D-glucose, pH 7.35) and transferred on ice to the cell-culture laboratory within one hour post-operation. The samples were finely cut. One part of each tissue sample was processed for magnetic bioseparation and the rest used for RNA and protein sample preparation as described below. The cut tumour tissue was washed twice in 1×PBS (PAA, Austria) and resuspended in stem cell buffer with added 1.33 mg/mL trypsin, 0.67 mg/mL hyaluronidase and 0.20 mg/mL kinurenic acid (all Sigma-Aldrich, Germany). After 90 min of incubation at 35°C, 5 % CO_2_, >95 % relative humidity with shaking, the samples were centrifuged for 10 min at 300×g (20°C). The supernatant was removed and the tissue resuspended in DMEM/F12 medium (PAA, Austria), supplemented with 0.7 mg/mL ovomucoid (Sigma-Aldrich). The tissue was finely dissociated with a thin glass Pasteur pipette and the tissue suspension was filtered through a 40 μm nylon mesh (BD Falcon, USA).

### Isolation of CD133+ glioblastoma cells

The single-cell suspension obtained was ten fold diluted in erythrocyte lysis-buffer (155 mM NH_4_Cl, 10 mM KHCO_3_, 0.1 mM EDTA), incubated for 5 min at 20°C, and centrifuged for 10 min at 300×g (20°C). The supernatant was removed and the cells resuspended in MACS buffer (1×PBS supplemented with 0.5 % w/v BSA and 2.0 mM EDTA, pH 7.2).

The prepared erythrocyte-free tumour cell suspension was used for direct magnetic bioseparation of CD133+ cells with miniMACS System (Miltenyi Biotec, Germany) according to the manufacturer’s protocol. The cell suspension was labelled with CD133+ MicroBeads in the presence of Fc reagent (both Miltenyi Biotec) for 30 min at 4°C, then diluted in 10×V MACS buffer, centrifuged for 10 min at 300×g (20°C), and resuspended in 500 μL MACS buffer. The cell suspension was applied to a magnetic separation column in a magnetic miniMAC-S™ Separation Unit. The CD133 negative fraction was eluted into a centrifuge tube without applying pressure. The separation column was then removed from the magnetic unit and the CD133+ fraction retained in the column washed out by applying pressure into a separate tube. The percentage of CD133+ cells in tumour samples was calculated as the ratio of the number of cells in the CD133+ fraction to the sum of the cells in the two fractions.

The efficacy of the separation was estimated by quantitative RT-PCR for CD133 as described below. Only separations in which the expression of CD133 mRNA was significantly higher in the CD133 positive fraction than in the negative fraction (F≥1.50) were considered successful, and taken into the study.

### Primary cultures of GBM biopsy

Primary cultures of unsorted cells and their CD133- cell populations from GBM samples 071017A and 071115 were prepared and grown as monolayers in DMEM/F12 medium, supplemented with 10 % foetal bovine serum, 4 mM L-glutamine, 1 % penicillin/streptomycin (all PAA) and 1M HEPES (Sigma-Aldrich). GBM spheroids were prepared from human glioblastoma biopsies as described elsewhere.^32^ Tumour biopsies were finely cut, resuspended in an appropriate volume of medium and seeded on agar coated cell culture dishes in a complete cell culture medium containing DMEM High Glucose (4.5 g/l), supplemented with 10 % foetal bovine serum, 1 % penicillin/streptomycin, 4 mM L-glutamine (all PAA) and 0.4 mM NEAA (Sigma-Aldrich). When the majority of the spheres reached 200 μm, they were dissociated by addition of 0.25 % trypsin -EDTA (Invitrogen, USA). The cell suspension was centrifuged for 10 min at 300×g, 10°C. The supernatant was removed and the cells distributed to new dishes in 1:3 dilutions. These cells started to form spheres after approximately 24 h.

### Normal neural stem cells (NNSC)

Neural stem cells were grown from subventricular zones of brain tissue collected *post-mortem* as described.^33^ The collection and use of brain tissue was approved by the Medical Ethics Committee of the Republic of Slovenia (156/07/09). The tissue samples were finely cut, degraded by trypsin (0.13 % w/V in water, Sigma-Aldrich) for 30 min at 37°C. Degradation was then blocked by 1 % foetal bovine serum in DMEM medium (both PAA). The tissue suspension was filtered through 40 μm nylon mesh, centrifuged for 5 min at 300×g, and resuspended in 10 mL of neurobasal medium, supplemented by basic fibroblast growth factor bFGF (20 ng/mL), EGF (20 ng/mL), serum supplement B27 (all Invitrogen, USA), heparin (1U/mL; Sigma-Aldrich), 1 % penicillin/streptomycin and 4 mM L-glutamine (both PAA). NNSC were grown in the form of spheres on non-adhesive culture dishes (Sarstedt, Germany).

### Established cell lines

Normal human astrocytes (NHA cells) and human glioblastoma cell line U87-MG were obtained commercially from Cambrex (USA) and American Type Culture Collection, respectively. Both cell lines were grown in monolayers in DMEM High Glucose (4.5 g/l), supplemented with 10 % foetal bovine serum, 1 % penicillin/streptomycin and 4 mM L-glutamine (all PAA). The NHA medium also contained 20 mM HEPES (Sigma-Aldrich, Germany). Cells were harvested by 0.25 % trypsin-EDTA (Invitrogen).

In addition we made use of two GBM stem-like cell cultures that were previously established at the Division of Neurosurgical Research, Heidelberg, Germany. These CD133+ GBM stem cell spheroids were grown from cells NCH644 and NCH421k, obtained as described by Campos *et al.*^34^, on non-adhesive culture dishes (Sarstedt) in serum-free DMEM/ F12 medium, 1 % penicillin/streptomycin and 4 mM L-glutamine (all PAA), supplemented by bFGF (20 ng/mL) and EGF (20 ng/mL) (both Invitrogen, USA), and BIT-supplement (Provitro, Germany).

All cells and spheroids were cultured under standard conditions at 37°C in humidified atmosphere with 5 % CO_2_. Unless otherwise specified, plastic-ware was purchased from Corning Costar Corporation, USA.

### 2D invasion assay of monolayers in Boyden chambers

Cells were tested for their invasion potential in a two dimensional invasion assay as described previously.^29^ Transwell chambers (Corning) with 8 μm pores were coated on the upper surface with Matrigel (0.25 mg/mL, BD Bioscience, USA) and 10^5^ cells were seeded. Fibronectin and conditioned serum free medium were used as chemoatractants. After 21 h incubation, MTT (1-(4,5-dimethylthiazol-2-yl)-2,5-diphenyl tetrazolium bromide, (Sigma-Aldrich) at 0.5 mg/mL final concentration was added to each chamber. After 3 h at 37°C, the formazan crystals that formed were collected separately from the upper and lower chambers, pelleted and dissolved in dimethyl sulphoxide, and the absorbance at 570 nm (reference filter 690 nm) measured on a spectrofluorimeter (Tecan). The percentage of cells penetrating Matrigel (invasive cells) was calculated as the ratio of the number of cells in the lower compartment to the sum of cells in both compartments. Invasion was normalised to that of normal neural stem cells NNSC.

### 3D invasion assay of spheres

Spheroids of NNSC, CD133+GBM stem cells NCH644 and NCH421k and GBM biopsy spheroids were tested for proliferation and invasive potential as described previously.^25^ Spheres (150–300 μm in diameter) were embedded in 50μL drops of type I collagen matrix (1.0 mg/mL, BD Bioscience). After incubating for 30–45 min at 37°C, the collagen was covered with cell culture medium. The spheroid diameter and cell invasion distance were measured under a light microscope using an ocular micrometer. Invasion distance was defined as the distance from the edge of the spheroid to the population of the cells most distant from the spheroid. Invasion was monitored for up to 21 days. Cell culture medium was changed every 3 days.

### DQ collagen degradation

Matrix degradation is one of the important features of the invasion process. To test the ability cells and spheroids to degrade the extracellular matrix, fluorescently labelled type IV collagen (DQ collagen IV, Invitrogen) was added to a Matrigel matrix (8.5 mg/mL). The spheroids were imbedded into 50 μL drops of Matrigel with 1 % DQ collagen IV as for the 3D invasion assay, whereas the cells grown in monolayer were plated on Lab-Tek Chamber Slides (Nunc, USA) pre-coated with Matrigel mixed with 2.5 % DQ collagen IV. After 24 h incubation, the green fluorescence of degraded DQ collagen IV was observed under a Zeiss LSMS10 confocal microscope. To visualise the cells/spheroids the visual light pictures of the same areas were superimposed.

### Quantitative real-time PCR

Samples were homogenized in TRIzol reagent (Invitrogen) and RNA isolated as suggested by the manufacturer. 1.0 μg of each RNA sample was reverse transcribed to cDNA using High Capacity cDNA Reverse Transcription Kit (Applied Biosystems, USA) following the manufacturer’s protocol.

Quantitative real-time-PCR assays were performed on ABI Prism 7900 HT Sequence Detection System using TaqMan Universal PCR Master Mix. Human GAPDH was used as internal control (all Applied Biosystems). Forward, reverse primers and probes sequences (in this order) were as follows: for CatB: 5′-CTC TATg AAT CCC ATg Tag ggT gC-3′, 5′-CCT gTT TgT Agg TCg ggC Tg-3′ and 5′-CCC TgT gAg CAC CAC gTC AAC gg-3′; for CatL: 5′-TCA ggA ATA Cag ggA Agg gAA A-3′, 5′-TCC Tgg gCT TAC ggT TTT gA-3′ and 5′-CAC Tgg TCA TgT CTC CAA Agg CgT TCA T-3′; for StefA: 5′-ggA ggC TTA TCT gAggCC AAA-3′, 5′-CAA gCT gTg gTT TAA CCT TAT CAA CA-3′ and 5′-CCg CCA CTC CAg AAA TCC Agg AgA-3′; for StefB: 5′-gCC gAg ACC CAg CAC ATC-3, 5′-ggC CTT AAA CAC Agg gAA CTT CT-3 and 5′-ACC Agg TgA ggT CCC AgC TTg AAg AgA-3; for CysC: 5′-gAC AAC TgC CCC TTC CAT gA-3, 5′-gCA CAg CgT AAA TCT ggA AAg A-3 and 5′-CAg CCA CAT CTg AAA Agg AAA gCA TTC Tg-3. The probes were 5′-FAM 3′-TAMRA modified. For CD133 (PROM1), Hs00195682_m1 and for CatS, Hs00175403_m1 TaqMan Gene Expression Assays (both Applied Biosystems) were used.

Due to the low expression of the CD133 mRNA, the pre-amplification step was performed before quantitative RT-PCR using the PreAmp Master Mix (Applied Biosystems) as suggested by the manufacturer.

mRNA data were calculated as 2^−ΔΔCt^ values. Fold differences in mRNA expression levels (F) between CD133+ and CD133− populations were calculated as in Demuth *et al.*^35^, where F = 2^(ΔCtCD133+ – ΔCtCD133−)^ and Ct_CD133+_ = Ct_GAPDH_ – Ct_target gene_ in the CD133+ cell in the population, and Ct_CD133−_ = Ct_GAPDH_ – Ct_target gene_ CD133− cell population. F ≥ 1.50 is considered as significantly higher and F ≤ 0.75 lower expression of the selected gene in CD133+ cells. F values between 1.50 and 0.75 are regarded as non-significant differences.

### Protein extraction and enzyme activity assays

Cells, grown in spheres were homogenized by sonication for 2 min in 50 mM Tris buffer, pH 6.9, supplemented with 0.05 % (V/V) Brij 35, 0.5 mM dithiothreitol, 5 mM EDTA, 0.5 mM paramethylsulphonyl fluoride and 10 mM pepstatin A (all Sigma-Aldrich). The homogenates were centrifuged for 30 min at 12.000×g (4°C) and the supernatants stored at −80°C until used.

CatB and CatL activities were measured as described previously.^25^ Duplicates of water diluted protein samples were supplemented with activation buffer (0.4 M phosphate buffer pH 6.0, 2.5 mM fresh ditiothreitol for CatB; 0.34 M acetate buffer, pH 4.2, 2.0 mM fresh DTE for CatL; all Sigma-Aldrich) and incubated for 30 min at 37°C. To measure specific cathepsin activity, water was added to one of the duplicates and specific inhibitor (60μM Ca-074, Peptide Institute, Japan, for CatB; 2μM Clik 148, provided by N. Katunuma, Tokushima Bunri University, Tokyo, Japan, for CatL) to the other. Activity buffer (0.4 M phosphate buffer pH 6.0, 2.5 mM fresh DTE for CatB; 0.34 M acetate buffer pH 5.5, fresh 2.5 mM DTE for CatL; all Sigma-Aldrich) was then added and the reaction started by adding specific substrate (100 μM Z-RR-AMC for CatB, 100 μM Z-FR-AMC for CatL; both Bachem, Switzerland). After 90 min at 37°C the reaction was stopped with 1 mM iodoacetic acid and the released 7-AMC measured on a spectrofluorimeter (Tecan). Specific cathepsin activity was calculated as the difference in 7-AMC release in the presence and the absence of the specific cathepsin inhibitor.

CatS activity was measured as described by Flannery *et al*.^30^ Water diluted protein samples were supplemented with inactivation buffer (100 mM phosphate buffer, pH 7.5) for 60 min at 37°C to fully inactivate CatB and CatL. The pH was then returned to 6.0 using 500 mM MES buffer. Reaction buffer (200 mM MES, 200 mM EDTA, pH 6.0, fresh 1 mM DTT, all Sigma-Aldrich) was added and the reaction started by adding specific substrate (100μM Z-VVR-AMC, Peptide Institute). After 90 min at 37°C the reaction was stopped with 100 mM acetate buffer, pH 4.3 and the released 7-AMC measured on a spectrofluorimeter.

Each activity assay was performed in triplicate, with controls with omitting the sample. Specific activities were expressed in enzyme units (E.U.) per mg of total protein, with one E.U. being the amount of the enzyme releasing 1 nm of 7-AMC per minute.

### Statistical analysis

All statistical analysis was performed with Excel 2002 (Microsoft Corp., USA) and Prism 5.01 (GraphPad Software Inc., USA). The statistical significances of the differences observed were calculated as standard t-test with assumed two-tailed distribution and unequal variance. For correlation studies, Spearman’s nonparametric method was used. Prognostic impact of CD133 expression for patient survival was calculated by relating it to overall survival by the Kaplan-Meier univariate analysis. To assess the association between survival period (from initial operation of the tumour to death of the patient) and other variables the Gehan-Breslow-Wilcoxon test was used.

## Results

### Prognostic value of CD133 mRNA in human GBM

**FIGURE 1.**
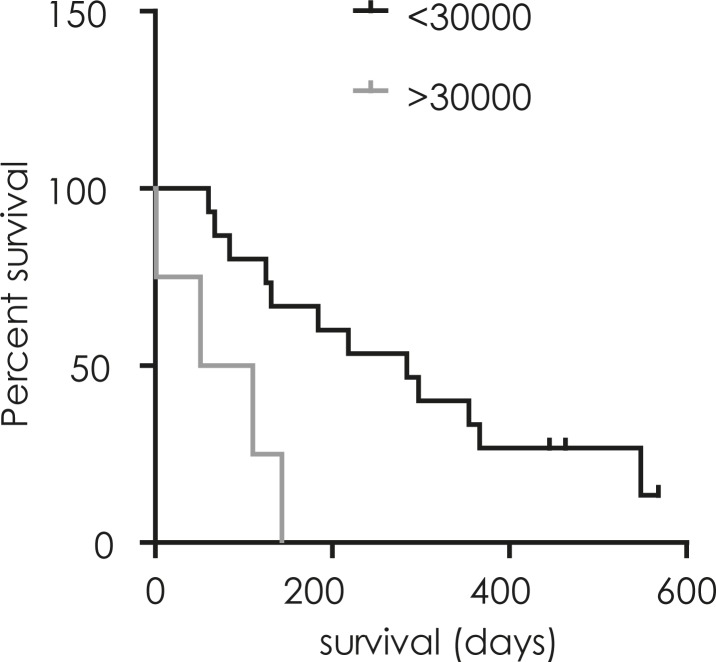
Prognostic impact of CD133 mRNA level on survival of the patients. CD133 mRNA expression was measured by QRT PCR in glioma samples of 19 patients and compared to their survival time post-operation. Survival of patients with higher CD133 mRNA levels above a cut-off of 30 000 (─, 2^−ΔΔCt^ > 30.000) was significantly shorter (median 81 days) than survival of patients with CD133 mRNA levels below the cut off (─, 2^−ΔΔCt^ < 30.000; median 284 days, p = 0.005).

Patient characteristics and survival after the operation are summarised in Table 1. 24 patients were diagnosed with WHO grade IV glioblastoma. In 19 of these, CD133+ mRNA expression in GBM tumour samples was compared with overall survival to assess the prognostic impact of CD133 mRNA (Figure 1). Survival of patients with higher CD133 mRNA levels (2^−ΔΔCt^ > 30.000) was significantly shorter (median 81 days) than that of patients with lower CD133 mRNA levels (median 284 days, p 0.005).

### Separation of CD133+ and CD133− cell populations from GBM samples

The cell suspensions prepared from GBM samples were subjected to direct magnetic bioseparation. In 15 samples the separation was successful, based on the difference in the CD133 mRNA expression being at least 1.5 time higher (F≥1.50) in the CD133+ than in the CD133^−^ fraction (see Materials & Methods). Only such samples were included in the further analysis. The abundance of the CD133+ cell fractions ranged from 2.0 % to 38.8 % of atotal cell population in individual tumour samples with an average of 9.6 ± 9.5 %.

### Comparison of CD133 with other tumour markers at mRNA levels

In mRNA extracts of CD133+ vs CD133− cells from 13 GBM patients we also compared the expression of other genes characteristics of neural and glioma cancer stem cells and their progenitors, such as nestin, and the markers for more differentiated cells, such as glial fibrillar acidic protein (GFAP) indicating astrocyte lineage differentiation, and β-tubulin 3 (β-TUB 3), the marker of neural differentiation. Table 2A shows that there was no correlation of their expression with CD133 within the group of samples.

### Comparison of CD133 with cathepsin expression at the mRNA level

In mRNA extracts of CD133+ vs CD133− cell fractions from 13 GBM patients we also measured mRNA levels of CatB, CatL, CatS, StefA, StefB and CysC (Table 2B). There was no significant correlation between these values. However, we observed that, in more than half the samples, the ratios of expression of CD133+ to CD133^−^ are lower (F≤1) in the three cathepsins. A similar trend was observed for the stefins, but an opposite one for cystatin C, for which increased levels in CD133+ samples were observed. This indicates that expression of the cathepsins increases with differentiation of CD133+ stem cells into the mature GBM cells.

### Cathepsin activity in CD133+ and CD133− cell populations

**FIGURE 2.**
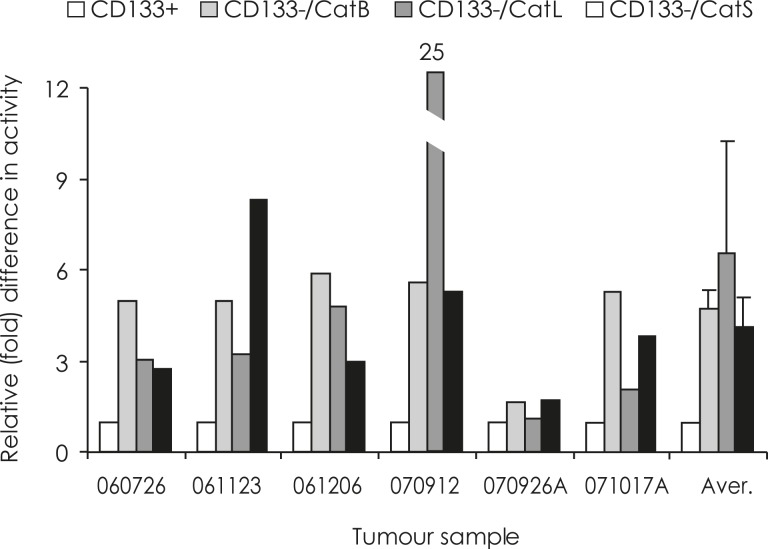
**Differences in cathepsins’ activities in CD133+ and CD133− cell fractions.** In each of the six GBM samples, a relative activity of 1 was assigned to all the CD133+ cell fractions (white bars). Fold differences in activity of cathepsins between CD133^−^ and CD133+ cell fractions were calculated as described in Material and Methods. CatB activity (light grey bars) was 1.7–5.9 times (average 3.9) higher, CatL activity (dark grey bars) 1.1–25 times (average 2.6) higher and CatS activity (black bars) 1.7–8.3 times (average 3.2) higher in the CD133− cell fractions than in CD133+ fractions.

In 6 tumour samples sufficient cellular material was obtained from the successful separation of CD133+ and CD133− cell fractions to assay cathepsin activities. In all the samples, CD133+ cell fractions contained significantly lower CatB, CatL and CatS activities than the CD133− cell fractions (Figure 2).

In parallel to their mRNA expression, the activities of CatB and CatL were higher, by 25 % and 37 %, in the spheroids of NNSC than in GBM stem cells NCH644.

### Relative expression of cathepsins and CD133 in various cell lines *in vitro*

**FIGURE 3.**
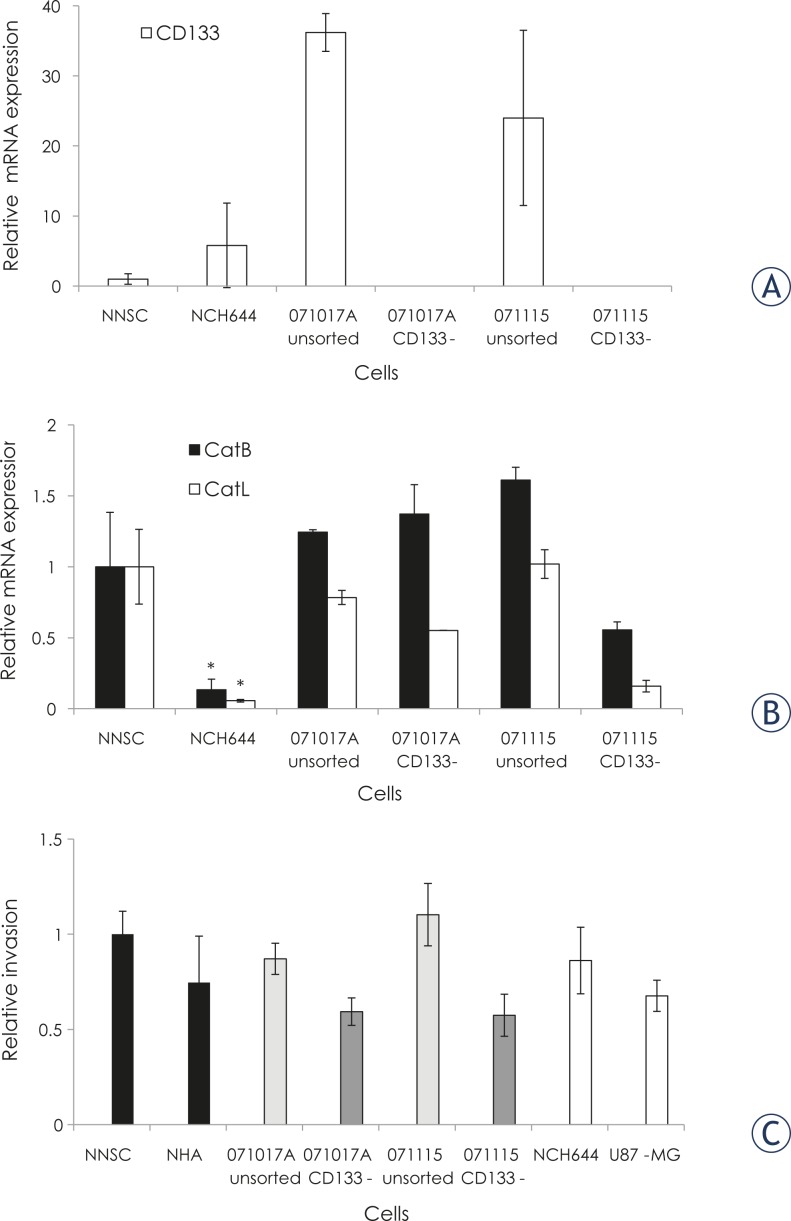
**Correlation between expression of cathepsins and 2D invasion in Matrigel. CD133 mRNA expression *in vitro***. CD133 mRNA expression was determined by QRT PCR in NNSC and NCH644 spheroids, and in unsorted GBM samples and in CD133− fractions from same GBM samples. A relative mRNA expression of 1 was assigned to NNSC. Cancer stem cells NCH644 cells were positive for CD133, however CD133 expression in (unsorted) GBM neurospheres was significantly higher (Student *t*-test, p<0.05) in spite of high variability between the three independent cell cultures, whereas in CD133− fractions from the same tumours, CD133 mRNA was below the detection limit. **Cathepsin B and Cathepsin L mRNA expression *in vitro***. Cathepsin B and Cathepsin L mRNA expression was determined by QRT PCR in NNSC and NCH644 spheroids, and unsorted GBM samples and CD133^−^ fractions from the same GBM samples. Relative mRNA expressions of 1 were assigned to NNSC. Cancer stem cells NCH644 cells expressed CatB and CatL at a significantly lower level than the NNSC (Student *t*-test, p<0.05). **Two dimensional cell invasion.** Two dimensional cell invasion of NNSC, NHA, unsorted GBM cells and CD133^−^ GBM cell populations, NCH644 and U87-MG cells, into Matrigel was carried out as described in Material and Methods. The percentage of invasive cells in NNSC was adjusted to 1 and other values were expressed as invasion relative to NNSC cells. The high variability in three independent experiments is reflected by relatively high inter assay S.D. values, whereas within each experiment the S.D. was always less than 10 %.

Due to the very high variability of CD133 mRNA measurements, the difference between its concentrations in the spheroids of NNSC and NCH644 cells was not significant. In primary cell cultures of two GBM samples, CD133 mRNA expression was similar in unsorted GBM cell populations, but below the limit of detection in CD133− cells remaining after CD133+ cell separation (Figure 3A).

CatB and CatL mRNA expression are presented in Figure 3B. NNSC expressed 7-fold and 18-fold higher levels of CatB and CatL than the GBM stem cells NCH644. The expression of Cats B and L in primary GBM samples was similar to or nonsignificantly higher than that in NNSC. However, there was no consistent difference in cathepsin expression in unsorted GBM and CD133^−^ cells from the same GBM samples.

### Invasion assays

#### 2D invasion assay

Normal NNSC and NHA cells and the GBM cells NCH644 and U87-MG, unsorted, and their respective CD133− cell populations from GBM samples, were tested for invasive potential in a two dimensional (Boyden chamber) invasion assay. The results were normalised to the invasiveness of NNSC (Figure 3C). Unsorted GBM cells were always more invasive than the CD133− cell population from the same tumour. Interestingly, NNSC exhibited higher invasive potential than the GBM stem cells CD133+ NCH644, and NHA appeared to be more invasive than the malignant U87-MG cells, although the differences were not significant. These data correlate approximately with the expression of Cats B and L (Figure 3B).

#### 3D invasion assay in collagen type I

**FIGURE 4.**
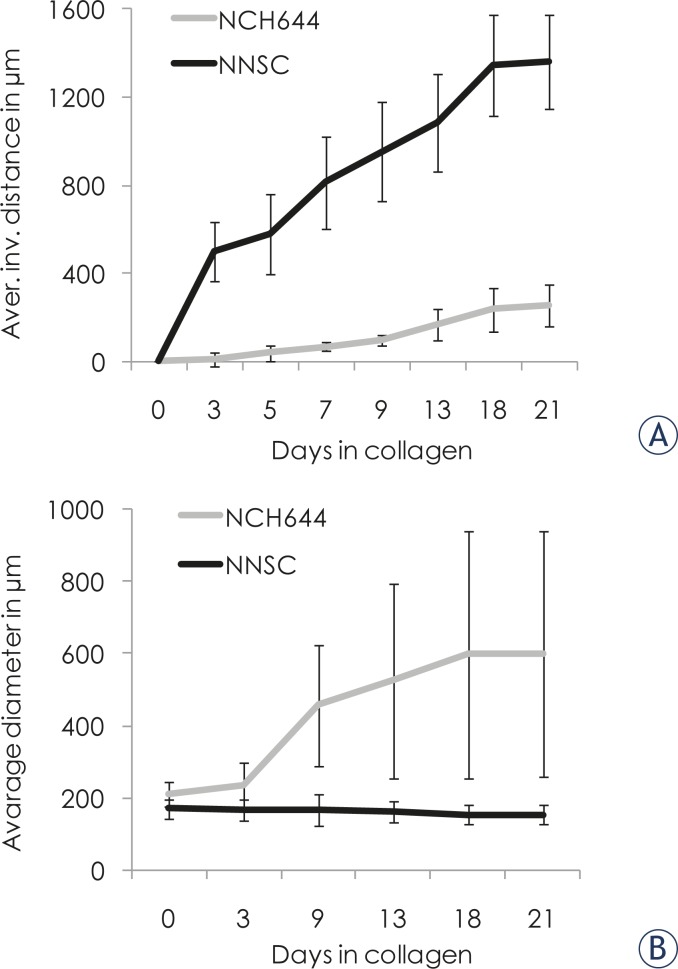

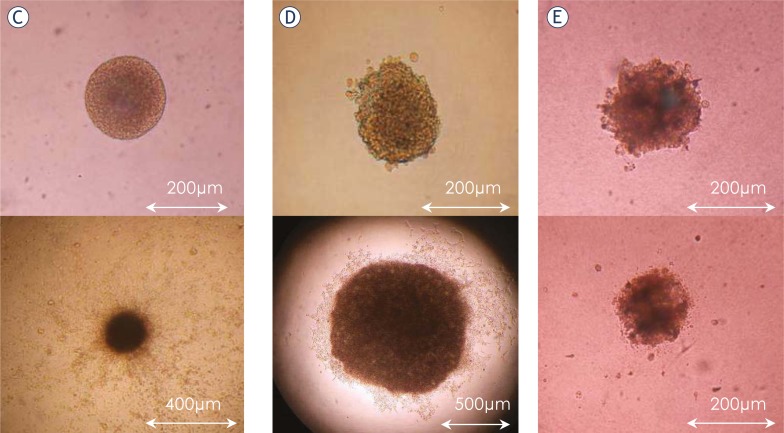
**3D spheroid invasion assay.** Spheroids were imbedded into collagen I and the invasion distance (panel A) and diameter (panel B) measured under the light microscope for up to 21 days. The average invasion distance was significantly higher (p<0.05) for spheres of NNSC than for spheres of NCH644. The average spheroid size did not change significantly in NNSC, but increased (p=0.009) in spheres of GBM stem cells, NCH644. GBM biopsy spheroids did not change in size and very few cells invaded the surrounding collagen. Panels C, D and E show the spheroids of NNCS, NCH644 cells and GBM spheroid, respectively at the start (upper panel) and after 21 days (lower panel) of the experiment.

The invasion and proliferation of the spheroids of NNSC, GBM stem cells and GBM biopsy spheroids were monitored in a 3D assay (Figure 4). The mode of invasion of NNSC spheres differed from that of the GBM stem cells. The average invasion distance after 21 days in collagen was 1359±216 μm for spheres of NNSC and 253±95 μm for spheres of GBM stem cells (Figure 4A). The NNSC proliferate slowly, with the size of their spheroids decreasing slightly throughout the experiment (from 170±26 μm to 153±27 μm, Figures 4B and C). In contrast, the spheroids of GBM stem cells NCH644 and NCH421k all grew in size from about 200 μm to an average of 598±340 μm (Figure 4B and D). The high S.D. was due to the variation observed among individual spheroids; about half of them grew to a diameter of over 800 μm in 21 days, whereas the others appeared to proliferate only in the first week of the experiment. The invasiveness of GBM biopsy spheroids appeared very limited; only a few cells invaded the surrounding collagen matrix, and we observed almost no change in spheroid size (Figure 4E). Due to adhesive interactions, GBM spheroids, containing a heterogeneous cell population, were less invasive than established cultured cells. Taken together, these data show that cell proliferation is higher in cancerous cells, and that invasion and proliferation appear to be inversely correlated.

### DQ collagen degradation

**FIGURE 5.**
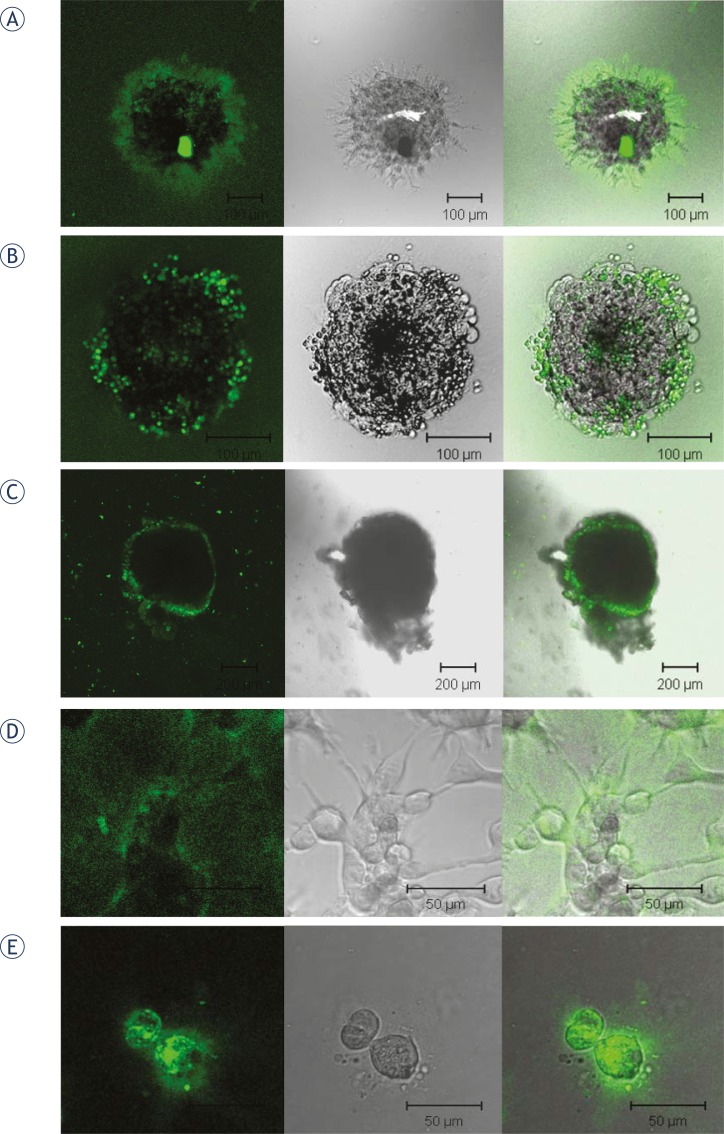
**Matrix (DQ collagen) degradation by neurospheres and cells grown in monolayers.** 1 % (for neurospheres) and 2.5 % (for the permanent cell lines grown in monolayers) DQ collagen type IV was added to Matrigel and matrix degradation observed after 24 h. Left: green fluorescence of degraded DQ collagen under Zeiss LSMS10 confocal microscope. Middle: visual light images of the same areas. Right: green fluorescence and visual light images combined. A: Spheroid of normal neural stem cells NNSC, B: Spheroid of GBM stem cells NCH644, C: GBM spheroid, D: U87-MG cells and E: NHA cells.

The ability of NNSC, GBM stem cells, GBM biopsy spheroids, and the permanent cell lines U87-MG and NHA to degrade the extracellular matrix was demonstrated by breakdown of DQ collagen, monitored by green fluorescence (Figure 5). In spheroids of NNSC, green fluorescence, indicating matrix degradation, was dispersed radially from the sphere centre, most propably along the migration paths of the cells leaving the sphere (Figure 5A). In spheroids of CD133+ GBM stem cells, the cells had not migrated from the sphere after 24 hours, and matrix degradation was localized to small areas on the surface of the sphere (Figure 5B). In GBM biopsy spheroids, the green fluorescence was dispersed over the whole surface of the spheroid (Figure 5C). When grown in monolayers, fluorescence was less intense and more dispersed in GBM cells U87-MG (Figure 5D) than in the normal human astrocytes NHA (Figure 5E). This shows that, in spite of significantly different patterns and rates of invasion between normal and cancer SC and between more differentiated normal and cancer cells, the process involves proteolysis. This may be more extracellular in normal neural cell spheroids and to a larger extent intracellular in tumour cells at the GBM spheroid surface.

## Discussion

Transmembrane protein CD133, alone or in combination with other markers, is the most common marker of stem cells in glioma. In this study, CD133+ cells were isolated from the most malignant brain tumour glioblastoma (GBM). As in other studies^36^,^37^, rather variable amounts of these cells were obtained, ranging from 2 % to 38 %. Further, we confirmed that the survival of GBM patients with higher CD133 mRNA expression was significantly shorter than in those with lower CD133 levels. Zeppernick *et al*.^38^ also found that both the proportion of CD133+ cells and their topological organization using immunohistochemitry (IHC), were prognostic factors for adverse, progression-free survival and that the proportion of CD133+ cells was an independent risk factor for GBM regrowth. In a prospective study of GBM patients, it was demonstrated that CD133+/Ki67+ was a considerable prognostic factor of disease progression and poor clinical outcome.^39^ High CD133 expression in high-grade oligodendroglial tumours was reported to indicate shorter survival and to be more reliable than histological assessment.^13^ Although it was postulated that CD133 could not be evaluated so accurately by real-time PCR^40^ as by IHC, in our hands mRNA CD133 levels had a prognostic impact similar to that of CD133 protein expression in the above studies. We believe that the reliability was due to inclusion of a pre-amplification step in cell mRNA analysis because of the low levels of CD133 transcript.

Comparing CD133 expression with other differentiation markers and markers of lysosomal proteolysis in CD133+ cells, such as nestin and CatB, no correlation in the cohort of 13 GBM patients was found. In our previous studies nestin and Cat B correlated and were both highly prognostic, as proven by IHC^23^,^41^ and mRNA analysis.^24^ This indicates, therefore, that CD133 is a prognostic factor independent of nestin and cathepsins, indicating its specific biological impact on survival.

It has been postulated that cancer stem cells can develop a migratory phenotype and are responsible for the metastatic potential of tumours, such as colon carcinoma.^8^ In this study we questioned whether CD133+ stem cells are also responsible for the high invasiveness of GBM. Two dimensional (2D) invasion assays showed that the invasion of various normal and cancerous cells was not correlated to their expression of CD133. Being aware of the limits of 2D invasion assay^25^,^42^, we also monitored invasion distance and sphere diameter in a three dimensional (3D) invasion assay on spheres of NNSC, NCH644, NCH421k and GBM biopsy spheroids. Normal neurospheres were undoubtedly more invasive than the spheres of GBM stem cells. However, the latter were more proliferative, since the sphere diameter tripled within the first week of experiment, while it was shrinking in the spheres of NNSC. GBM spheroids also showed a more limited invasive potential than U87-GM spheroids^25^, most probably due to the heterogeneous cell population within tumour samples, with higher intercellular adhesion. In our hands, we found higher migratory potential of normal than cancerous spheroids and this was inversely related to CD133 expression, suggesting that its expression does not play a role in cell invasion.

However, according to a current hypothesis^15^, only tumour stem cells (also called tumour initiating cells) are capable of tumour renewal, therefore they must acquire migratory properties. Migratory stem cells were clearly visualised in the 3D assays and these cells may represent the invasive malignant GBM cell phenotype. However, there was no evidence that the migratory cell subpopulation of CD133+ cell and GBM biopsy spheroids still expressed this marker. High plasticity of GBM tumour initiating (stem) cells with respect to CD133 expression was suggested, as not only CD133+^11^,^38^,^43^ but also CD133− spheroids^15^,^32^ were tumorigenic in animals. On the other hand, U87 cells which, when treated with neural stem cell medium, altered their phenotype towards more stem like cells, increasing the levels of CD133 and nestin, and induced highly infiltrative tumours in animals.^44^ A model has been proposed in which CD133^+^ cells constitute a non-invading GBM SC population with the potential to switch reversibly between an invasive and stationary phenotype. This involves an epithelial to mesenchymal transition, followed by a mesenchymal to epithelial transition when seeded to the secondary site.^8^ This transition, associated with reversible loss and gain of CD133 marker and possibly associated with a set of migratory proteins like Cats (B, S), is an attractive hypothesis that could explain our results.

Here we have demonstrated for the first time that radial cellular migration from the spheres is accompanied by proteolysis of DQ collagen. Invasion of the cells was therefore associated with the activation of proteases required for matrix degradation. Presumably, cathepsins are involved in the initial steps of a proteolytic cascade^45^, leading to pericellular proteolysis, although alternative pathways of migration and interplay of proteases and inhibitors are possible.^18^,^46^,^47^ In high grade tumours higher expression of Cats B and S has been mostly linked to tumour invasion.^18^,^19^,^22^,^30^,^48^ We recently reported higher levels of all three Cats in invading than in non-invading cells separated from collagen embedded U87-MG spheroids, however only increased activation of CatB contributed to higher invasion.^25^ Here, significantly higher Cats levels were observed in the more migratory NNSC than in GBM NCH644 cells at mRNA levels. Normal neurospheres appear to migrate by extracellular dissolution of collagen matrix, whereas its degradation is to a greater extent intracellular within tumour cells, as suggested also for other tumor cells previously^49^ when compared with normal neural cells. This suggests that different modes of invasion may be associated with the different proteolysis pathways activated in normal and tumour cell migration.^46^

Inverse correlation of CD133 with invasion (Figure 3) corresponds to our clinical data, where we showed no correlation of Cat mRNA levels with CD133 and lower Cat activity in CD133+ cell fractions from GBM tumours. Cat activation in CD133− cells may be explained by their up-regulation and/ or the downregulation of their inhibitors, such as cystatin C and/or stefins (A and B)^48^, during the process of GBM stem cell differentiation. Similarly, CatB was reported to be upregulated after the differentiation of monocytes into tissue macrophages, but not earlier in hematopoetic differentiation^49^ and CatL expression was upregulated during angiogenesis from endothelial progenitor cells.^50^ These results suggest that GBM stem cells are not as invasive as their progenitors, losing CD133 and acquiring migratory properties by activation of a set of proteolytic enzymes, including Cats.

In conclusion, this study confirms that CD133 is a prognostic marker for survival of GBM patients. We have demonstrated that NNSC have higher invasion potential and invade the collagen matrix in a mode which differs from that of GBM initiating stem spheres. This result could have implications for designing new therapeutics, including protease inhibitors that may be specifically delivered by novel technologies, developing for drug delivery, to target invasive tumour stem cells. Increased expression of cathepsin activities in CD133 negative cells suggests their role in the invasive GBM stem cell progenitors.

## Figures and Tables

**TABLE 1.** Patient characteristics, therapy and overall survival

**Tumour sample**				**Patients**	

NIB No.	histopathol. diagnosis	Gender	Age	Survival (days)	Additional therapies
AA 060726	AA	female	58	51	NAT
AA 080424	AA	female	34	*463	ChT/RT + ChT + DITEM
GBM 061017	GBM	male	57	548	ChT/RT + ChT
GBM 061123	GBM	female	68	175	ChT/RT
GBM 061206	GBM	male	80	215	NAT
GBM 070103	GBM	male	37	366	ChT/RT + ChT + BCNU
GBM 070322	GBM	female	74	84	NAT
GBM 070402	GBM	male	74	60	NAT
GBM 070904	GBM	female	70	273	ChT/RT
GBM 070912	GBM	male	58	218	ChT/RT + ChT
GBM 070926A	GBM	male	65	67	unfinished RT
GBM 070926B	GBM	female	60	271	ChT/RT
GBM 071017A	GBM	male	43	297	ChT/RT + ChT + c.pr.
GBM 071017B	GBM	male	27	284	ChT/RT + ChT
GBM 071115A	GBM	male	48	371	ChT/RT + ChT + BCNU
GBM 080107	GBM	male	68	110	palRT
GBM 080110	GBM	male	45	*568	ChT/RT + ChT + BCNU
GBM 080129	GBM	female	78	125	NAT
GBM 080512	GBM	female	72	*445	palRT
GBM 080521	GBM	male	66	131	ChT/RT + ChT + BCNU
GBM 080528	GBM	male	50	143	ChT/RT
GBM 080603	GBM	male	43	184	ChT/RT + ChT + AVA
GBM 080612	GBM	male	78	354	ChT/RT
GBM 080619	GBM	male	60	1	NAT
GBM 090909	GBM	female	50	110	palRT
GBM 090921	GBM	female	59	*394	ChT/RT + ChT + DITEM

**Histopathological diagnosis of the tumours:** AA- anaplastic astrocytoma (WHO grade III), GBM- glioblastoma (WHO grade IV).

**Age:** age of the patients at the time of the operation in years.

**Survival:** survival of the patients after the first operation in days (*- the patients were still alive at the end of data collection).

**Additional therapy** (all the patients were operated, most also received additional therapies): NAT - no additional therapy used, ChT/RT- standard combination of chemotherapy (temozolomide) and radiotherapy (60 Gy), ChT- standard chemotherapy repeated, BCNU- additional chemotherapy with bis-chloronitrosourea, c.pr.- complementary medicine program, palRT- palliative radiotherapy, DITEM- dose dense chemotherapy with temozolomide, AVA- additional chemotherapy with avastin, palRT- palliative radiotherapy.

**TABLE 2. A:** Correlation between CD133 and other differentiation markers

**Tumour** Samples (n=13)	**F**			
	CD133	nestin	GFAP	TUB3
GBM 061123	**3,19**	1,46	**0,66**	**1,66**
GBM 061206	**32,9**	**2,27**	**2,48**	0,92
GBM 070103	**2,65**	**2,03**	**1,53**	1,36
GBM 070402	**3,43**	**0,65**	**0,20**	0,98
GBM 070904	**6,82**	**0,59**	1,22	**0,46**
GBM 070912	4,85	2,80	3,21	0,69
GBM 070926A	**2,34**	1,09	1,36	1,08
GBM 070926B	**1,66**	**0,70**	**0,68**	1,13
GBM 071017A	**3,67**	1,02	**2,02**	1,42
GBM 071017B	5,14	5,72	3,48	2,05
GBM 071115A	**5,55**	**2,18**	**4,74**	0,91
GBM 080110	**5,60**	0,94	**1,98**	**0,12**
GBM 080512	**2,11**	**1,99**	1,46	0,80

**TABLE 2. B:** Correlation between CD133 and expression of cathepsins and stefins

Tumour Samples (n=13)	**F**						
	CD133	CatB	CatL	CatS	StefA	StefB	CysC
GBM 061123	3,19	0,40	0,13	0,20	**0,09**	**0,61**	1,33
GBM 061206	**32,9**	**1,60**	**2,50**	**3,87**	**1,73**	1,35	1,28
GBM 070103	**2,65**	0,88	0,92	1,00	0,76	**0,69**	**0,68**
GBM 070402	**3,43**	1,14	**0,73**	**0,43**	1,09	**0,71**	**0,44**
GBM 070904	**6,82**	**0,15**	**0,18**	**0,03**	*	**0,45**	*
GBM 070912	4,85	0,11	0,14	0,08	**0,06**	**0,32**	0,90
GBM 070926A	**2,34**	1,03	0,76	**0,74**	**0,70**	0,86	0,92
GBM 070926B	**1,66**	1,39	1,16	0,99	1,24	0,93	1,26
GBM 071017A	3,67	0,61	0,33	0,10	0,08	0,51	1,57
GBM 071017B	**5,14**	**4,50**	**3,09**	**2,32**	1,38	**2,28**	**3,29**
GBM 071115A	**5,55**	**0,32**	**0,42**	**0,28**	*	0,85	*
GBM 080110	**5,60**	1,14	**0,61**	**0,45**	**0,31**	0,90	**2,87**
GBM 080512	**2,11**	**0,36**	**0,67**	**1,81**	**0,41**	1,30	0,78

The relation factor F represents the ratio of the expression of mRNA of the CD133/prominin 1 to that of other markers in a and to cathepsins B, L and S and stefins A and B in b in CD133 positive and negative cells. The cells were separated from primary GBM by magnetic separation, as described in Material and methods. All significantly altered values are in bold: F ≥1.50 means significantly higher levels of expression in CD133^+^ cells, F≤0.75 means signficantly lower expression in CD133^+^ cells.

(*) means that the levels were below the limit of detection.
